# Socioeconomic and psychological correlates of postpartum depression at 6 months in Dhaka, Bangladesh

**DOI:** 10.1002/ijop.12735

**Published:** 2020-12-16

**Authors:** Viviane Valdes, Anne E. Berens, Charles A. Nelson

**Affiliations:** ^1^ Boston Children's Hospital Harvard Medical School, Labs of Cognitive Neuroscience Boston USA; ^2^ Harvard Graduate School of Education Cambridge MA USA

**Keywords:** Postpartum depression, Prevalence, Psychological, Socioeconomic

## Abstract

To current study aimed to estimate the point prevalence and identify correlates of postpartum depression (PPD) in a sample of mothers in Dhaka. A total of 235 participants from low‐ and middle‐SES neighbourhoods in Dhaka completed the Edinburgh Postnatal Depression Scale (EPDS) and other assessments of socioeconomic and psychological factors at 24 weeks postpartum. Regression models were fit to explore potential correlates of PPD. The estimated prevalence of high PPD risk in the current sample is 24.3%. In multivariable linear regression models, recent life events, perceived stress and household resources (e.g., access to cooking gas, telephone, furniture, electricity, television, etc.) were significantly associated with PPD. The association of social support with PPD when controlling for other variables was sensitive to the choice of social support measure, highlighting an important methodological issue. The point prevalence of PPD among poor, urban mothers in Bangladesh ranges from 12.3 to 28.5%, with psychological risk factors and household resources as strong correlates.

Depression affects an estimated 121 million people globally, making it the second leading contributor to years lived with disability (YLDs) (Reddy, 2010; WHO, 2017). The World Health Organization (WHO) estimates that in high‐income countries (HIC), 13% of women experience a mental disorder in the first postpartum year (Hendrick et al., 1998; WHO, 2017). In low‐ and middle‐income countries (LMIC) PPD prevalence is estimated at approximately 20% according to meta‐analyses examining PPD at various timepoints postpartum (Fisher et al., 2012; Hendrick et al., 1998). Like major depressive disorder, postpartum depression (PPD) is characterised by two of three major criteria: dysphoria, anhedonia or decreased energy in addition to four additional symptoms over the course of two or more weeks, between 4 and 30 weeks after childbirth (Andrews‐Fike, 1999; APA, 2013).

Despite a potentially higher burden among mothers in LMIC, PPD is often understudied in these regions. Only 17/112 WHO prevalence estimates for common mental health disorders after birth are from LMIC (Fisher et al., 2012). Furthermore, it is unclear whether findings about risk and protective factors from HIC are generalizable to LMIC where the burden of PPD could be experienced differently, and treatment may be less available. In Bangladesh, specifically, the point prevalence of antepartum depression in a rural sample is estimated at 18% and the point prevalence of PPD in a clinical convenience sample (*n* = 100) was estimated at 9% at 6–8 weeks postpartum (Gausia et al., 2007; Nasreen et al., 2011).

There are several known risk factors for PPD, most of which have been established from research in HIC. In an earlier meta‐analysis of risk factors for PPD (O'hara & Swain, 1996), the strongest correlates of PPD (Cohen's *d* ≥ 0.8) include depression or anxiety during pregnancy, stressful life events during pregnancy or early puerperium, low levels of social support, or a history of pre‐pregnancy depression, findings since well‐supported by additional studies (Fisher et al., 2004; Fisher et al., 2010; Paulson et al., 2006; Rahman et al., 2003). Moderate risk factors in the above meta‐analysis for PPD (Cohen's *d* ≥ 0.4 and < 0.8) include childcare stress, low self‐esteem in mothers and difficult child temperament (O'hara & Swain, 1996). Weak risk factors (Cohen's *d* ≥ 0.2 and < 0.4) include obstetric or pregnancy complications, negative cognitive attributions, poor relationship with partner or presence of abuse and socioeconomic status (SES) (O'hara & Swain, 1996).

Interventions to reduce PPD may not only relieve suffering for mothers but also have substantial benefits for children and families given well‐documented links between maternal depression and child development outcomes (Drewett et al., 2004; Mondal et al., 2009; Patel et al., 2004; Paulson et al., 2006). Examining the point prevalence of PPD and initial exploration of factors associated with PPD risk thus may have substantial clinical and public health importance in the study context.

The current study estimates the point prevalence of maternal PPD 24 weeks after birth in a mixed‐income sample in Dhaka, Bangladesh. It then examines the potential psychological and socioeconomic correlates of PPD. Analyses consider hypotheses that: (i) Psychological and socioeconomic risk factors are independently associated with PPD risk, (ii) associations of socioeconomic factors with PPD risk will be attenuated when accounting for psychological risk factors and (iii) context‐tailored measures of psychological risk factors may provide greater sensitivity than widely‐used measures originally developed in HIC contexts.

## METHODS

### Sample

Analysis here focuses on infant‐mother dyads (*n* = 235) recruited into the Bangladesh Early Adversity Neuroimaging (BEAN) study and assessed at 6 months postpartum. The sample included participants from low‐SES neighbourhoods (*n* = 130) who were recruited from the longitudinal Burden of Cryptosporidiosis Study, which was originally developed by door‐to‐door invitation of all eligible mothers Mirpur. Additional middle‐SES participants in the current sample (*n* = 105) were recruited by door‐to‐door visiting of houses in a middle‐SES neighbourhood nearby the Mirpur district and inviting all age‐appropriate mothers to participate. For recruitment in both low‐ and middle‐income SES neighbourhoods, study workers went door‐to‐door to all homes and identified households with mothers at the appropriate age range (0–24 weeks postpartum) and had infants born at ≥36 weeks of gestation. Mothers were excluded from the study if their child had a history of known neurological abnormalities, genetic disorders, or visual/auditory impairments. Once households with eligible mothers were identified, study workers visited their homes again to tell mothers about the study and invite them to participate in a full study visit at the International Centre for Diarrhoeal Disease Research, Bangladesh (ICDDR,B). Sample size calculations suggested that for a mild effect size of 0.10, at a statistical power level of 0.8, a probability level of .05 and with 10 predictors in linear regression, a total sample size of 172 participants would be necessary. Of 475 eligible mothers approached to participate in the study, 235 enrolled in the study and 240 refused to participate (49.37%).

### Study context

The BEAN Study assesses various child developmental risk factors, including psychosocial risks such as PPD (see measures), as well as development outcomes using behavioural scales and neuroimaging assays. It represents a collaboration between Boston Children's Hospital/Harvard Medical School, University College London, University of Virginia and ICDDR,B with funding from the Bill and Melinda Gates Foundation.

### Procedures

PPD point prevalence and risk factors were estimated using data collected at BEAN study visits 6 months postpartum. Mothers completed questionnaires on psychosocial risk factors in interview format (given low literacy) and responded to questions about socioeconomic characteristics, and infants underwent developmental assessments using behavioural scales and neuroimaging. All data were collected in private study clinic rooms after informed written consent with ethical approval from institutional review boards at ICDDR,B and Boston Children's Hospital.

### Measures

*Postpartum depression* was measured using the 10‐item Edinburgh Postnatal Depression Scale (EPDS), which has been validated as a clinical screening tool to identify PPD in Bangladeshi women at 6–8 weeks postpartum (Cox et al., 1987; Gausia et al., 2007). The WHO reports that there are no differences in the pooled mean point prevalence estimates of PPD for self‐reported symptom measures and diagnostic assessments, suggesting that self‐report methods are capable of generating reliable data (Fisher et al., 2012). EPDS score were generated as item score sums. The American Academy of Paediatrics and the only existing validation study in Bangladesh recommend a cutoff of 10 or greater (USA: 80% sensitivity, 92% specificity; Bangladesh: 89% sensitivity, 87% specificity) (Cox et al., 1987; Gausia et al., 2007). Recent research in US contexts (2014) indicates that a cutoff score greater than or equal to 9 increases sensitivity to 90% and specificity to 85% (Venkatesh et al., 2014). Prior studies, both in the USA and internationally, (2006) have used a suggested a cutoff as high as 13 (Matthey et al., 2006). Given the varying standards in cutoffs, all three cutoffs will be reported.

*Socioeconomic factors* were collected by parent report and included presence of household domestic workers, husband's level of education, maternal/own level of education, husband's occupation, maternal/own occupation, total monthly household income, total monthly household expenditure, household resources and neighbourhood SES. See Table 1 for an overview of how each variable was measured and operationalised.

**Table 1:** Descriptive statistics

Variable name	Mean (SD)	Range	Response category prevalence
Postnatal depression score (EPDS)	5.85 (4.99)	0–23	*Cutoff 9*: High likelihood of depression (HLD): 67 (28.5%); Low likelihood of depression (LLD): 168 (71.5%) *Cutoff 10*: HLD: 57 (24.3%); LLD: 178 (75.7%) *Cutoff 13*: HLD: 29 (12.3%); LLD: 206 (87.7%)
Household has hired domestic workers	—	—	No: 199 (84.7%); Yes: 26 (11.1%); Missing: 10 (4.3%)
Husband's level of education	—	0–5	0. No formal education: 42 (17.9%) 1. Some primary school (5th grade or less): 28 (11.9%) 2. Primary school certificate (5th grade): 35 (14.9%) 3. Junior school certificate (8th grade): 53 (22.6%) 4. Secondary school certificate (10th grade): 20 (8.5%) 5. Higher secondary school certificate or more (12th grade or higher): 47 (20%) Missing: 10 (4.3%)
Own level of education (maternal)	—	—	0. No formal education: 34 (14.5%) 1. Some primary school (5th grade or less): 31 (13.2%) 2. Primary school certificate (5th grade): 58 (24.7%) 3. Junior school certificate (8th grade): 47 (20%) 4. Secondary school certificate (10th grade): 19 (8.1%) 5. Higher secondary school certificate or more (12th grade or higher): 36 (15.3%) Missing: 10 (4.3%)
Husband occupation	—	—	‐Beggar or unemployed: 1 (0.4%) ‐Home skill worker, garment worker, unskilled labourer, sweeper, helper, retired: 44 (18.7%) ‐Student, landlord, tailor, cook, contractor, small business owner: 47 (20%) ‐Government service, NGO, medium businessman, teacher: 116 (49.4%) ‐Large business, doctor, engineer, lawyer: 17 (7.2%) ‐Missing: 10 (4.3%)
Maternal occupation	—	—	‐Unemployed: 3 (1.3%) ‐Student: 9 (3.8%) ‐Housewife: 197 (83.8%) ‐Employed: 16 (6.8%) ‐Missing: 10 (4.3%)
Total monthly income	Taka: 31,410.67 (23,543.53) USD: 377.24 (282.76)	Taka: 3000‐130,000 USD: 36.03–1561.30	—
Household size	5.71 (2.48)	3–15	—
Number of household resources^a^	8.70 (1.93)	2, 12	Median is a score of 9: 34 (14.5%)
Neighbourhood SES cohort	—	—	Low SES neighbourhood: 130 (55.3%) Middle SES neighbourhood: 105 (44.7%)
Recent life events questionnaire score	5.08 (2.86)	0, 17	—
Perceived Stress Scale score	15.31 (8.47)	0, 38	—
Multidimensional Scale of Perceived Social Support score	Mean raw total score: 57.67 (11.40), moderate support	21, 84	—
Social Support Score, CPAI‐B (middle‐class neighbourhood group only, *n* = 102)	1.84 (3.35)	0–14	—

EPDS = Edinburgh Postnatal Depression Scale; SES = Socioeconomic status; CPAI‐B = Childhood Psychosocial Adversity Index‐Bangladesh; NGO = Nongovernmental organisation.

^a^ One point for each of the following: cooking gas, telephone, almeria, table, chair, bench, watch/clock, electricity, cot/bed, radio, TV, bicycle, motorcycle, sewing machine, fan.

*Psychological factors* included exposure to stressful recent life events (RLE) measured using the RLE Questionnaire (reliability, α = .86) (Brugha et al., 1985; Cohen et al., 1991), stress perception measured using the Perceived Stress Scale (reliability, α = .85) (Cohen et al., 1991), and social support perception using the Multidimensional Scale of Perceived Social Support (MSPSS) (reliability, α = .88) (Zimet et al., 1988). The MSPSS has not been validated in Bangladesh. Consequently, further sensitivity analyses will be conducted with sub‐scales of the Childhood Psychosocial Adversity Scale—Bangladesh (CPAS‐B) among the middle‐SES cohort (the CPAS‐B was not created at the time of data collection for the low‐income sample). This questionnaire was developed specifically to ascertain aspects of psychosocial adversity, including social isolation (reliability, α = .93) and maternal depression (reliability, α = .96), for Bangladeshi samples based on substantial qualitative work and psychometric analysis (Berens et al., 2019).

### Statistical analysis

Statistical analysis was carried out using Stata Statistical Software Version 15 (College Station, TX, StataCorp LLC). Initial exploratory analysis included the generation of descriptive statistics to assess for non‐normality and outliers, and of a pairwise correlation matrix for all independent variables and our dependent variable of interest (continuous EPDS score). Descriptive statistics included point prevalence estimates for PPD. Next, ordinary least squares (OLS) linear regression models were built to test the four hypotheses.

Before conducting analyses, data were cleaned to account for missing values. First, participants who were missing all SES variables or all psychological variables were dropped (*n* = 9). Sixty‐five participants (60 from the low SES cohort) were missing information regarding RLE. A *t*‐test for those missing RLE data compared to those not showed significant higher perceived stress (3.33 points, *p* = .03). Multiple imputations were used for all psychological factors to preserve variability. For all other remaining values, simple mean imputation was used to handle missing data. See Table 1 for the specific number of observations imputed by variable.

For bivariate and multivariate analyses, PPD was modelled using EPDS score as a continuous variable, since cutoff standards for binary categorizations vary widely. Prevalence estimates are reported for cutoff scores of 9, 10 and 13 to allow comparability with existing research.

To test whether psychological risk factors were associated EPDS scores, regressions were built including psychological risk factors as independent variables, modelling EPDS score as the dependent variable. The same procedure was repeated for SES‐related independent variables. As observational data do not allow for clear demonstration of mediation relationships, we undertook preliminary exploration of our hypothesis that psychological factors may mediate the relationship between SES and PPD using a hierarchical linear regression model whereby SES variables were included first followed by psychological risk factors.

We then tested the hypothesis that neighbourhood SES may interact with psychological factors by replicating all factor regression models that include relevant interaction terms between all psychological factors and neighbourhood SES. Finally, we tested whether the social isolation sub‐scale of the CPAS‐B is a more sensitive correlate of PPD among participants from middle‐ SES neighbourhoods compared to the MSPSS.

*Compliance with Ethical Standards*: All procedures performed in studies involving human participants were in accordance with the ethical standards of the institutional research committee and with the 1964 Helsinki Declaration and its later amendments or comparable ethical standards.

*Informed Consent*: Ethics approval was obtained from Boston Children's Hospital Institutional Review Board (IRB‐P00015728) and International Centre for Diarrhoeal Disease Research, Bangladesh Institutional Review Board (PR‐14110). Informed consent was obtained from all participants.

## RESULTS

### Sample characteristics

Mean maternal age was 23.66 (*SD* = 3.13) among low‐SES neighbourhood participants, ranging from 18 to 35. Mean monthly household income among low‐SES neighbourhood participants was 15,149.23 taka (*SD* = 9612.86) or 181.94 USD while that of middle‐SES neighbourhood participants was 51,543.87 taka (*SD* = 19,818.25 taka) or 619.04 USD (*t* = −18.43, *p* < .0001). Household per capita income was estimated at 2988.85 taka or 36 USD in low‐SES neighbourhoods and 8885.27 taka or 107.01 USD in middle‐SES neighbourhoods (*t* = −15.48, *p* < .0001). The modal education level for husbands was 8th grade (53, 22.6%) and 42 husbands (17.9%) had no formal education. Considering maternal education, the modal education level was 5th grade (58, 24.7%) and 34 mothers (14.5%) had no formal education. There were differences between paternal versus maternal education (*χ* ^2^ = 172.48, Cramer's *V* = 0.36, *p* < .0001), with husbands, on average, having more education (Table 1).

Recruited districts appear to be representative of low‐ and middle‐income populations in Bangladesh based on two key characteristics: income and maternal education. UNICEF reports that the average annual per capita income in Bangladesh is $840 USD or 70,486 Taka, which monthly translates to $70 or 5874 Taka per capita (Barkat et al., 2015; UNICEF, 2012). In our sample, the low‐income group had an average monthly household income of $181.94 USD or 15,149.23 Taka while our middle‐income group had an average annual household income of $619.04 USD or 51,543.87 Taka. In terms of education, UNICEF suggests that 50.6% of females enrolled in secondary school. In our low‐income sample, we found that only 10.8% (14/130) got at least a secondary school certificate (10th grade and higher) while in the middle‐income group, 43.2% (41/95) did (Barkat et al., 2015; UNICEF, 2012).

### Point prevalence of postpartum depression

In the current study, using a cutoff score of 10 on the EPDS, 57 (24.3%) participants were identified as having a high likelihood of depression (≥10 on the EPDS) at postpartum week 24, while 178 (75.7%) participants were identified as having a low likelihood of depression (score ≤ 9 on the EPDS). Among the low SES neighbourhood group, using the same threshold, 45/130 (34.6%) participants were identified as having a high likelihood of depression while in the middle SES group only 12/96 (12.5%) had a high likelihood of depression. Point prevalence estimates for different EPDS cutoff points can be found in Table 1.

### Bivariate analyses

In bivariate analysis, EPDS score (continuous variable, with higher scores indicating higher risk) was significantly correlated with socioeconomic variables including: the presence of household domestic workers (no, yes) (*r* = −.21, *p* < .001), husband's level of education (ordinal) (−0.18, *p* = .007), maternal education (ordinal) (−0.17, *p* = .013), husband's occupation (ordinal) (−0.14, *p* = .032), monthly household income (continuous) (−0.17, *p* < .001), monthly expenditure (continuous) (−0.18, *p* = .008), monthly per capita income (continuous) (−0.19, *p* = .004), number of household resources (continuous) (−0.29, *p* < .001) and neighbourhood SES (−0.227, *p* < .0001). Here, higher SES across all variables was associated with lower EPDS scores. Stressful RLE (*r* = .38, *p* < .001) and perceived stress (0.49, *p* < .001) were positively correlated with EPDS. When measured using the MSPSS, maternal social support was significantly, negatively associated with EPDS scores (*r* = −.22, *p* = .001). In the middle‐class sub‐cohort (*n* = 102) that also completed the CPAI‐B, maternal social isolation (inverse of maternal social support) on the CPAI‐B was also significantly correlated with maternal depression (*r* = .5241, *p* < .001) while MSPSS score was not (*r* = .03, *p* = .78) (Table 2).

**Table 2:** Correlation coefficients for all variables—ordered socioeconomic, psychological and maternal age

	1	2	3	4	5	6	7	8	9	10	11	12	13
1. Postnatal depression	—												
2. Domestic household workers (yes/no)	−.21**	—											
3. Husband's level of education	−.178**	.35***	—										
4. Own level of education (maternal)	−.17*	.32***	.64***	—									
5. Husband's occupation	−.14*	.28***	.40***	.43***	—								
6. Own occupation (maternal)	.04	0	−.18**	−.09	−.08	—							
7. Monthly income	−.17**	.34***	.50***	.50***	.60***	−.19**	—						
8. Household resources	−.29***	.36***	.53***	.52***	.55***	−.22**	.66***	—					
9. Neighbourhood SES	−.27***	.36***	.55***	.52***	.58***	−.21**	.77***	.64***	—				
10. Recent life events	.38***	−.06	−.05	−.06	−.03	.10	−.14*	−.08	−.09	—			
11. Perceived stress	.49***	−.10	−.06	.04	−.03	.03	0	−.08	−.07	.31***	—		
12. Social support (MSPSS)	−.22**	.20**	.27***	.41***	.26***	−.11	.37***	.31***	.43***	−.09	−.09	—	
13. Maternal age	.10	.02	−.04	−.05	.10	−.01	.01	−.0	0	.10	.08	−.07	—

^*^ *p* < .05.

^**^ *p* < .01.

^***^ *p* < .001.

### Multivariate regression analyses

The first regression model assessing the significance of SES factors as correlates of PPD showed that household resources (*β*_*std*_ = −.26, *p* = .006) was the only variable associated with PPD (Table 3, model 1) in the overall sample. The model including only SES‐related exposures had an adjusted *R* ^2^ of .12. The next regression model to test for the predictive ability of psychological factors on PPD found that all of the psychological variables tested including stressful RLE (*β*_*std*_ = .24, *p* = .001), perceived stress (*β*_*std*_ = .38, *p* < .0001) and social support (*β*_*std*_ = −.13, *p* = .023) were significantly associated with EPDS scores and had an adjusted *R* ^2^ of .32 (model 2). In the combined model including SES and psychological variables (*n* = 235), RLE, perceived stress remained significant and household resources remain significant, while social support did not (model 3). No significant interactions between neighbourhood SES and psychological factors were found (model 4).

**Table 3:** Multivariate regression models—Edinburgh Postnatal Depression Scale score 6 months after birth (*n* = 235)

	Model 1 Socioeconomic factors only	Model 2 Psychological factors only	Model 3 All factors	Model 4 Accounting for interaction
Presence of domestic workers	−.12 −1.84 (1.12)		−.08 −1.19 (.95)	−.09 −1.32 (.95)
Husband's level of education	.02 .06 (.24)		.05 .12 (.21)	.05 .13 (.20)
Maternal level of education	−.007 −.02 (.27)		−.05 −.15 (.24)	−.05 −.14 (.24)
Husband's occupation	.05 .30 (.47)		.04 .24 (.41)	.05 .25 (.41)
Monthly income (1000‐taka units)	.16 .03 (.02)		.15 .03 (.02)	.14 .03 (.02)
Household resources	−.26** −.68 (.24)		−.21* −.55 (.21)	−.21* −.56 (.21)
Neighbourhood SES cohort	−.20* −2.06 (1.83)		−.16 −1.67 (1.04)	−.62* −6.26 (3.02)
Recent life events		.25** .36 (.10)	.24** .36 (.10)	.24** .35 (.10)
Perceived stress		.38*** .22 (.04)	.36*** .21 (.04)	.35*** .21 (.04)
Social support		−.13* −.06 (.03)	−.04 −.02 (.03)	−.14 −.06 (.04)
Social support by neighbourhood SES interaction				.51 .08 (.05)
Constant	11.04 (1.83)	3.94 (1.72)	5.89 (2.31)	8.34 (2.77)
*R* ^2^	.12	.32	.38	.38
Adjusted *R* ^2^	.09	.31	.35	.35
Df for model *F*	7	3	10	11
*F*	4.01***	29.16***	1.92***	11.13***

Reported in standardised β coefficients in first row and unstandardized β coefficients (standard error in parentheses) on second row.

^*^ *p* < .05.

^**^ *p* < .01.

^***^ *p* < .001.

In sensitivity analyses (Table 4), several differences were apparent. First, in the subset of 102 participants from the middle‐SES cohort that took both the psychological questionnaires used in prior models as well as the CPAS‐B (a context‐tailored tool generated for use in Bangladesh), maternal social support measured by the MSPSS was not significantly associated with EPDS scores (*β*_*std*_ = .03, *p* = .71, model 1). However, when the same model was fit including the CPAS‐B subscale score measuring maternal social isolation (inverse of social support) in place of the MSPSS, social isolation was significantly associated with EPDS scores (*β*_*std*_ = .39, *p* < .001, model 2). This model had an adjusted *R* ^2^ of .45.

**Table 4:** Effects of different psychosocial measures on regression model estimates in middle‐class cohort (*n* = 102)

	Postpartum depression measured by EPDS		Postpartum depression measured by CPAI‐B
	Model 1 (MSPSS)	Model 2 (CPAI‐B social isolation)	Model 3 (CPAI‐B social isolation)
Presence of domestic workers	−.14 −1.46 (.96)	−.15 −1.52 (.86)	−.02 −.31 (1.38)
Husband's level of education	.06 .17 (.28)	.03 .09 (.25)	.04 .18 (.40)
Maternal level of education	−.08 −.22 (.31)	.02 .05 (.28)	−.23* −1.15 (.45)
Husband's occupation	−.05 −.45 (.76)	.01 .11 (.70)	.08 1.07 (1.12)
Monthly income (1000‐taka units)	.23* .05 (.02)	.17* .04 (.02)	−.03 .01 (.03)
Household resources	−.06 −.22 (.38)	−.07 −.27 (.35)	.11 .72 (.55)
Recent life events	.29** .42 (.14)	.23* .33 (.12)	.31*** .75 (.21)
Perceived stress	.31** .16 (.05)	.21* .11 (.05)	.17* .15 (.08)
Social support (MSPSS)	.03 .01 (.03)		
Social isolation (CPAI‐B)		.39*** .50 (.11)	.43*** .93 (.18)
Constant	.61 (4.86)	.52 (3.99)	−7.46 (6.37)
*R* ^2^	.32	.45	.50
Adjusted *R* ^2^	.26	.39	.45
Df for model *F*	9	9	9
*F*	4.92***	8.25***	10.26***

Reported in standardised β coefficients in first row and unstandardized β coefficients (standard error in parentheses) on second row

^*^ *p* < .05.

^**^ *p* < .01.

^***^ *p* < .001.

In these models, there was a paradoxical positive correlation between household income and EPDS score, with a one standard deviation increase in household income being associated with a 0.23‐point increase in EPDS score (*β*_*std*_ = .23, *p* = .01). However, this paradoxical association was not evident in a final model including all prior factors but using the CPAS‐B to measure both maternal social isolation and PPD. In this model, an incremental move from a higher to lower level of maternal education (education strata are shown in Table 1) was associated with 1.15‐point increment in EPDS score (*β*_*std*_ = −.23, *p* = .01). Stressful RLE (*β*_*std*_ = .31, *p* < .001), perceived stress (*β*_*std*_ = .21, *p* = .04) and maternal social isolation (*β*_*std*_ = .39, *p* < .001) were also again significantly associated with higher EPDS score. This model, using context‐tailored measures of both maternal depression and social isolation, had an *R* ^2^ of .50.

## DISCUSSION

The point prevalence of women with PPD symptoms in the current sample from Dhaka, Bangladesh was 24.3% at 24 weeks postpartum according to the clinical screening cutoff score of 10 previously validated in Bangladesh (Azad et al., 2019; Gausia et al., 2007; Islam et al., 2017; Nasreen et al., 2015). Using the more stringent cutoff of 13, the point prevalence is lower at 12.3%, in line with the 13%‐point prevalence reported in meta‐analyses of international samples (predominantly in HIC, cutoff of 13) (O'hara & Swain, 1996). Given that the Bangladesh EPDS validation study indicated that a cutoff of 10 had the highest sensitivity/specificity, and based on recent recommendations (2014), future research should estimate the international prevalence of PPD using these lower cutoffs and include more LMIC since the burden of PPD may be higher than existing international meta‐analyses suggest (Cox et al., 1987; Venkatesh et al., 2014).

Regression models suggest that psychological factors independently may have a stronger association with PPD than SES‐related variables (accounting for 31 and 8% of the variance in EPDS scores, respectively) in the present sample. In the SES‐only model, household resources were the only significant correlate of lower EPDS scores, perhaps due to the more limited variability in SES in our sample (only low‐ and middle‐income families). Meanwhile, in the psychological factors‐only model, all of the variables tested were associated with EPDS scores (more RLE, perceived stress and less social support). However, it is important to note that due to the cross‐sectional nature of the current study, the use of observational data to characterise predictive factors does not allow us to support claims about causality or temporal relations between stress perception and PPD. Additional research is needed to better delineate the nature of the relation between the perception of stress and PPD.

When adjusting for SES, social support was no longer significantly associated with PPD when using the MSPSS, which is widely used internationally, but was developed for use in HIC and has not been validated in Bangladesh. Use of the CPAS‐B led to different conclusions; maternal social isolation remained highly associated with PPD symptoms and the explained variance in depression scores increased. The increased power of the CPAS‐B social isolation scale could reflect that social support and social isolation function differently, or differences in the sub‐sample that happened to complete both measures. However, this pattern of results is also consistent with decreased measurement error associated with using a locally‐tailored tool, and highlights the sensitivity of findings to the choice of psychological instrument. CPAS‐B all‐factor models also suggest that beyond social isolation, maternal education (protective), stressful life events and perceived stress were independently and significantly associated with maternal depression. Finally, no interactive effects were found between neighbourhood SES and social support on PPD.

The relationship between SES and PPD has been explored extensively in existing research. Studies in both LMICs and HICs suggest that low income, and SES more broadly, are significantly related to PPD (Mukherjee et al., 2017; Nasreen et al., 2015; O'hara & Swain, 1996; Verbeek et al., 2019). Recent research in rural and urban areas of Bangladesh also find that maternal education is a protective factor for PPD, with inverse associations between maternal education and PPD (Azad et al., 2019; Nasreen et al., 2015). Our study finds protective effects of education, household resources and neighbourhood income on postpartum depression. Research on the impact of stress perception suggests that higher levels of perceived stress increase risk for PPD (Azad et al., 2019; Islam et al., 2017; O'hara & Swain, 1996). The literature on the role of exposure to stressful life events is less consistent. RLEs are an established risk factor for PPD in western samples, but past studies in Japan and Hong Kong found that they were not associated with PPD (Lee et al., 2000; Zayas et al., 2003). The current study indicates the stressful life events may be a risk factor in the current Bangladeshi population. However, these findings may also be explained by reverse causality, with PPD resulting in higher levels of perceived stress due to cognitive distortions associated with depression. The protective influence of social support, especially perceived social support, on PPD has been well‐established in past research (Islam et al., 2017; Milgrom et al., 2019; Zayas et al., 2003). Our current analysis supports the importance of this variable, but highlights measurement issues that could influence conclusions about this and other psychosocial variables.

The current findings suggest that the point prevalence of women at high risk of PPD in the current sample may be between 12.3 and 28.5% depending on the cutoff utilised. Our findings also suggest that psychological risk factors such as stressful recent life events, perceived stress and social isolation may be more closely linked with PPD than socioeconomic factors despite challenges in measuring these variables. Among socioeconomic factors, household resources, maternal education and neighbourhood income are significantly related to PPD. When adjusting for SES, social support (using MSPSS) is no longer a significant protective factor for PPD, potentially due to a lack of local validation for this measure or other explanations discussed above. These findings extend past research in HICs suggesting that both psychological and socioeconomic factors confer risk for PPD to an urban area of Bangladesh. Our findings also suggest that in low‐income contexts other measures of wealth may be more appropriate to ascertain PPD risk than household salary (e.g., neighbourhood income level and household resources). These results provide early observational evidence that lower neighbourhood income, household resources, maternal education and higher stressful life events, perceived stress and social isolation may put Bangladeshi mothers at increased risk for PPD. This finding can help to inform future research, practice, interventions and policy in the region.

### Limitations

The present analysis has several limitations that should be considered. With regards to point prevalence estimates, a single EPDS score at 24 weeks risks missing cases that resolve before or develop after this time point (or the most severe depressive cases where mothers may commit suicide). It is also important to note that EPDS cutoffs for identifying PPD risk were developed for clinical screening, and not all mothers with a “high risk” EPDS score would necessarily meet diagnostic criteria; therefore, we cannot make claims about clinical PPD, only those at risk. The use of cross‐sectional data in the current study also does not allow us to support claims about causality or comment on other correlates of PPD explored in past research (e.g., history of mental illness, childcare stress, maternal self‐esteem and child temperament, teenage pregnancy) (O'hara & Swain, 1996). In addition, common method bias (correlation between two or more variables as a result of using a shared method to measure them) could account for a part of the associations observed, further supporting the need for future research using distinct methodologies to measure the psychological constructs of interest. Furthermore, while the validity of using EPDS scores to screen clinically for PPD has been explored previously in Bangladesh (Gausia et al., 2007), similar context‐specific analysis has not been performed to assess the validity of some of our measures of maternal social support, stressful life events and perceived stress. Finally, it should be noted that although the samples in the current study were obtained by convenience and may not be wholly representative of underlying populations, our middle‐income group is near national averages in income and education, while our low‐income group is well below.

These limitations notwithstanding, the relationships presented provide a foundation for ongoing research on risk and protective factors for PPD in Bangladesh that could be targeted in supportive interventions. Moreover, in the context of the BEAN project as a whole, PPD may prove to be an influential factor on infant outcome. Our study included several established variables and provides insight into how they may be associated with PPD in an urban setting in Bangladesh. Future research should assess for causal links and temporal relations in Bangladesh and aim to validate screening methods like the EPDS across various time points postpartum to maximise our ability to identify and intervene.

## Data Availability

The data that support the findings of this study are available from the Bill and Melinda Gates Foundation but restrictions apply to the availability of these data. Data are however available from the authors upon reasonable request and with permission of the Bill and Melinda Gates Foundation.
